# Pandemic Worry and Preventive Health Behaviors During the COVID-19 Outbreak

**DOI:** 10.3389/fmed.2021.700072

**Published:** 2021-06-17

**Authors:** Minghui Li, Gang Lv, Stephanie Hsieh, Rongjie Shao, Jing Yuan

**Affiliations:** ^1^Department of Clinical Pharmacy and Translational Science, University of Tennessee Health Science Center, Memphis, TN, United States; ^2^Department of General Surgery, The First Medical Center of Chinese PLA General Hospital, Beijing, China; ^3^Department of Pharmacy, Scarborough Health Network—Centenary Hospital, Scarborough, ON, Canada; ^4^Department of Health Economics, China Pharmaceutical University, Nanjing, China; ^5^Department of Clinical Pharmacy and Pharmacy Administration, Fudan University School of Pharmacy, Shanghai, China

**Keywords:** COVID-19, pandemic worry, hand washing, face mask wearing, maintaining social distance

## Abstract

**Objective:** As schools are preparing for onsite learning, it is urgently needed to characterize the extent of pandemic worry and to examine predictors of adopting preventive health behaviors of hand washing, face mask wearing, and maintaining social distance among student pharmacists.

**Methods:** An online survey was sent to 326 student pharmacists in the United States. Pandemic worry was measured using a seven-point Likert scale ranging from extremely not afraid of, to extremely afraid of getting COVID-19. The health belief model (HBM) was the theoretical framework of this study. Preventive health behaviors and components of the HBM were also measured using seven-point Likert scales (one indicated extremely unlikely; seven indicated extremely likely). Multivariable linear regression models were used to identify predictors of each behavior.

**Results:** A medium level of pandemic worry (*M* = 4.2, *SD* = 1.92) was identified and females reported a higher pandemic worry. Respondents reported that they were extremely likely to wash their hands (*M* = 6.8, *SD* = 0.48) and maintain social distance (*M* = 6.6, *SD* = 0.92), but were moderately unlikely to wear face masks (*M* = 2.2, *SD* = 1.51). Determinants of face mask wearing included pandemic worry, perceived benefits, cue to action, self-efficacy, and being of an Asian American. Perceived barriers were negatively associated with face mask wearing.

**Conclusion:** Strategies should be implemented to reduce the psychological impact of COVID-19 pandemic among student pharmacists. Predictors identified in this study should be incorporated in efforts to improve face mask wearing. Continued monitoring of pandemic worry and preventive health behaviors is of great significance when universities and colleges are for onsite learning.

## Introduction

Coronavirus disease 2019 (COVID-19) is a new respiratory illness caused by SARS-CoV-2 coronavirus. It was first detected in Wuhan, China in December 2019 and has rapidly spread worldwide (1). COVID-19 was declared as a pandemic by the World Health Organization (WHO) in March 2020 (2). The first US case of COVID-19 was reported in Washington State on January 19, 2020 (3). From the initial clusters identified in the states of Washington, California, and New York, confirmed cases of COVID-19 quickly expanded to all 50 states (4). The federal government implemented Coronavirus Guidelines and put forth certain preventative measures to slow the spread of COVID-19, such as maintaining good hand hygiene, avoiding social gatherings, closing schools, and non-essential businesses in areas with evidence of community transmission. Subsequently, multiple state and local officials implemented stay-at-home orders. Despite these containment efforts, the number of confirmed cases of COVID-19 continued to rise nationally (5). The United States has now the highest number of COVID-19 infections and has become the new epicenter of the pandemic (1).

The COVID-19 outbreak has generated significant pandemic worry. Pandemic worry refers to the constant, uncontrollable, and unwanted negative thoughts about the future outcomes of disease (6). These thoughts might impede individuals' problem-solving skills, prevent engagement in protective health behaviors, and contribute to the development of anxiety and depression. College students might be particularly vulnerable to the psychological impacts as they face pressure in many aspects of life, such as in academics and employment, especially given their immaturely developed psychological system. With the current COVID-19 outbreak, significant pandemic worry has been reported among college students in China (7, 8). This psychological impact might be due to the consequences of national and local measures taken during the outbreak, including school closures and rising unemployment rates secondary to lockdowns. Colleges and universities in the United States have implemented certain preventive measures, such as closures of campuses, cancellation of experiential rotations, changes in the delivery of didactic courses and exams, which might adversely affect the psychological well-being of college students (9). Negative psychological consequences due to the abruptness and timing of these implementations near the end of the academic school year include graduation delays, jeopardization of campus housing, and meal programs security, poor adaptation to the new learning style and exam delivery, and inability to continue with lectures for those with resource constraints.

Individuals' preventive health behaviors are important to contain the spread of COVID-19. The health belief model (HBM) is a theoretical framework that has been widely used in previous infectious disease outbreaks to evaluate individuals' motivations to adopt preventive health behaviors (10–14). Key components of the HBM include perceived susceptibility (how likely the individual perceives they will get the disease), perceived severity (individuals' perception of the severity of the illness), perceived benefits (individuals' belief about the effectiveness of the health behavior), perceived barriers (individuals' belief of the obstacles that prevent them from adopting the health behavior), cue to action (an external stimulus that triggers an individual to adopt the behavior), and self-efficacy (individuals' confidence of their ability to engage in the behavior) (15). In the 2002 to 2003 Severe Acute Respiratory Syndrome (SARS) epidemic, perceived susceptibility, perceived benefits, and cue to action significantly predicted face mask wearing and hand washing in Hong Kong (10, 11). In the 2009 H1N1 pandemic, perceived susceptibility, perceived severity, perceived benefits, and cue to action determined the adoption of maintaining social distance and hand washing in South Korea (12). For face mask wearing during the H1N1 pandemic, perceived susceptibility, perceived benefits, perceived barriers, and cue to action were significant predictors in Hong Kong (14).

To slow the spread of COVID-19, the Center for Disease Control and Prevention (CDC) has recommended that individuals adopt certain preventive health behaviors. These recommendations include hand washing with soap and water or hand sanitizer, keeping a six-feet social distance from other people, and covering the mouth and nose with a cloth face mask when around others (16). The guidance on face mask wearing was modified in April 2020 in light of recent evidence suggesting that transmission of the SARS-CoV-2 coronavirus might still occur among asymptomatic and pre-symptomatic individuals (17). Because these individuals comprise of a significant proportion of people infected with COVID-19, the CDC revised previous recommendation that face masks should only be worn by sick or caregivers of sick individuals, to the new recommendation that everyone should wear a cloth face mask in public settings when social distancing is hard to maintain.

To the best of our knowledge, no studies have evaluated the impact of pandemic worry on the adoption of preventive health behaviors during the pandemic of COVID-19 among student pharmacists. Health professional students are facing a high risk of COVID-19 infections through their interning, practicing, and working environments. To fill the gap in the literature, the objectives of this study were: (1) to characterize the extent of pandemic worry and (2) to examine predictors of adopting CDC-endorsed preventive health behaviors, including hand washing, face mask wearing, and maintaining social distance, among student pharmacists.

## Methods

An online survey was sent to 326 student pharmacists enrolled in a PharmD program in the south of the United States. Both those attended didactic course and engaged in experiential learning were surveyed in this study. A pilot study of five respondents was conducted before the formal study to make sure the clarity of survey questions. The survey was distributed in March 2020 and finished in 2 weeks. During that time, the number of COVID-19 cases continued to grow in the United States. Following the federal Coronavirus Guidelines and state and local stay-at-home orders, most colleges, and universities were closed and didactic courses were adjusted to virtual learning. In addition, preceptors at some clinical sites were unable to take student pharmacists who are considered non-essential. Thus, most clinical rotations were rescheduled or canceled.

Information collected in this study included demographics, academic setting, pandemic worry, knowledge, and preventive health behaviors. Demographic information collected included age, gender, and race. Didactic course and experiential learning were two academic settings studied. Pandemic worry was measured by frequency (seven-point Likert scale, from never to always worried about getting COVID-19) and severity (seven-point Likert scale, from extremely not afraid of to extremely afraid of getting COVID-19). Knowledge of COVID-19 was measured by the source of information, availability of test kit, medication, and vaccine, and perceived knowledge (seven-point Likert scale, from extremely low to extremely high).

CDC-endorsed preventive health behaviors, including hand washing, face mask wearing, and maintaining social distance, were examined in this study (16). The likelihood of performing these preventive health behaviors was reported using seven-point Likert scales, where one indicated extremely unlikely and seven indicated extremely likely. Key components of the HBM, including perceived susceptibility, perceived severity, perceived benefits, perceived barrier, cue to action, and self-efficacy, were also measured using seven-point Likert scales.

The distributions of demographics, academic setting, pandemic worry, and knowledge were reported by frequency and percentage. Means of pandemic worry, knowledge, and preventive health behaviors were analyzed and compared by different age, gender, race, and academic setting groups using *t*-tests or analysis of variance (ANOVA) tests. The correlations between different preventive health behaviors were measured using Pearson correlation coefficients. Multivariable linear regression models were used to identify predictors of hand washing, face mask wearing, and maintaining social distance. In each regression model, the preventive health behavior was the dependent variable and pandemic worry, knowledge, components of the HBM, demographics, and academic setting were independent variables. SAS version 9.4 (SAS Institute, Cary, NC) was used to perform all statistical analyses.

## Results

Among 326 respondents who completed the survey, the majority of them were aged 18–26 years (79%), female (74%), and Caucasian (68%). The distribution between different academic settings at the time of survey completion was approximately equal, including 53% attending didactic course and 47% engaged in experiential learning (Table 1). Most respondents reported hearing about COVID-19 through digital channels such as social media (73%), TV (43%), and websites (40%). Respondents reported a medium level of pandemic worry severity (*M* = 4.2, *SD* = 1.92). The availability of a test kit (92%), lack of availability of approved medications (74%), and lack of availability of a vaccine (93%) for COVID-19 were correctly identified by the majority of respondents (Table 1). Respondents reported a slightly high perceived knowledge of COVID-19 (*M* = 4.8, *SD* = 1.03). Females reported a significantly lower knowledge compared to males (4.7 vs. 5.2, *p* < 0.001), and those attended didactic course reported a significantly lower knowledge compared to those engaged in experiential learning (4.7 vs. 4.9, *p* = 0.03; Table 2).

**Table 1:** Demographics, academic setting, pandemic worry, and knowledge of study participants (*N* = 326).

	***N***	**%**
**Demographics**		
**Age**		
18–26	258	79.1
27+	68	20.9
**Gender**		
Female	242	74.2
Male	84	25.8
**Race**		
Caucasian	222	68.1
African American	40	12.3
Asian American	45	13.8
Other	19	5.8
**Academic setting**		
Didactic course	172	52.8
Experiential learning	154	47.2
**Pandemic worry**		
**Frequency**		
Never	30	9.2
Occasionally	74	22.7
Sometimes	63	19.3
About half the time	37	11.3
Often	59	18.1
Most of the time	41	12.6
Always	22	6.7
**Knowledge**		
**Source**		
TV	140	42.9
Website	129	39.6
Social media	239	73.3
Radio	22	6.7
In print	23	7.1
Family/friend	136	41.7
**Availability of test kit**		
Yes	299	91.7
No	17	5.2
Unsure	10	3.1
**Availability of medication**		
Yes	33	10.1
No	242	74.2
Unsure	51	15.6
**Availability of vaccine**		
Yes	5	1.5
No	303	92.9
Unsure	18	5.5

**Table 2:** Pandemic worry, knowledge, and preventive health behaviors of study participants by age, gender, race, and academic setting.

	**Total**	**Age**			**Gender**			**Race**					**Academic setting**		
		**18–26**	**27+**	***p* value**	**Female**	**Male**	***p* value**	**Caucasian**	**African**	**Asian**	**Other**	***p* value**	**Didactic**	**Experiential**	***p* value**
									**American**	**American**			**Course**	**Learning**	
	***M* (*SD*)^†^**	***M* (*SD*)**	***M* (*SD*)**		***M* (*SD*)**	***M* (*SD*)**		***M* (*SD*)**	***M* (*SD*)**	***M* (*SD*)**	***M* (*SD*)**		***M* (*SD*)**	***M* (*SD*)**	
**Pandemic worry**	4.2 (1.92)	4.2 (1.93)	4.3 (1.90)	0.50	4.4 (1.87)	3.7 (1.96)	0.004^*^	4.1 (1.92)	4.1 (2.00)	4.8 (1.83)	4.5 (1.81)	0.13	4.2 (1.88)	4.2 (1.97)	0.85
**Knowledge**	4.8 (1.03)	4.8 (1.00)	4.7 (1.16)	0.33	4.7 (1.02)	5.2 (0.99)	<0.001^*^	4.9 (0.96)	4.7 (1.09)	4.5 (1.18)	4.6 (1.34)	0.14	4.7 (1.04)	4.9 (1.02)	0.03^*^
**Preventive health behavior**															
Hand washing	6.8 (0.48)	6.8 (0.43)	6.8 (0.62)	0.53	6.9 (0.44)	6.7 (0.56)	0.006^*^	6.8 (0.50)	6.9 (0.35)	6.8 (0.53)	6.9 (0.32)	0.34	6.8 (0.48)	6.8 (0.48)	0.51
Face mask wearing	2.2 (1.51)	2.1 (1.45)	2.3 (1.74)	0.37	2.2 (1.49)	2.2 (1.59)	0.89	2.1 (1.46)	2.0 (1.27)	2.9 (1.91)	2.1 (0.97)	0.004^*^	2.2 (1.51)	2.1 (1.52)	0.72
Maintaining social distance	6.6 (0.92)	6.6 (0.94)	6.6 (0.85)	0.86	6.6 (0.91)	6.5 (0.96)	0.20	6.6 (0.98)	6.5 (1.06)	6.8 (0.47)	6.5 (0.70)	0.46	6.6 (0.79)	6.5 (1.05)	0.16
**Perceived susceptibility**	4.7 (1.79)	4.7 (1.81)	4.8 (1.75)	0.64	4.7 (1.80)	4.8 (1.77)	0.59	4.8 (1.73)	4.6 (1.84)	4.0 (1.94)	5.5 (1.54)	0.006^*^	4.4 (1.87)	5.1 (1.62)	<0.001^*^
**Perceived severity**	5.8 (1.13)	5.8 (1.08)	5.9 (1.29)	0.80	5.9 (1.07)	5.7 (1.27)	0.17	5.8 (1.12)	5.9 (1.03)	6.0 (0.95)	5.4 (1.64)	0.27	5.8 (1.19)	5.9 (1.05)	0.17
**Perceived benefits**															
Hand washing	6.0 (1.01)	6.1 (1.01)	5.9 (1.00)	0.27	6.1 (1.00)	5.9 (1.02)	0.11	6.0 (0.99)	6.2 (1.08)	6.0 (1.00)	6.1 (1.18)	0.85	6.0 (1.04)	6.1 (0.98)	0.83
Face mask wearing	3.5 (1.71)	3.5 (1.68)	3.8 (1.81)	0.24	3.6 (1.70)	3.3 (1.73)	0.09	3.5 (1.72)	3.6 (1.75)	3.9 (1.76)	3.3 (1.33)	0.43	3.4 (1.67)	3.6 (1.75)	0.29
Maintaining social distance	6.2 (0.97)	6.3 (0.94)	6.1 (1.06)	0.13	6.3 (0.96)	6.1 (0.98)	0.12	6.2 (0.99)	6.2 (1.11)	6.5 (0.73)	6.5 (0.77)	0.19	6.3 (0.95)	6.2 (0.98)	0.20
**Perceived barrier**															
Hand washing	3.4 (2.04)	3.4 (2.05)	3.3 (2.04)	0.56	3.4 (2.03)	3.5 (2.09)	0.72	3.5 (2.01)	3.0 (2.17)	3.7 (2.10)	2.7 (1.89)	0.18	3.3 (1.97)	3.6 (2.12)	0.23
Face mask wearing	4.9 (1.88)	4.9 (1.87)	4.8 (1.93)	0.91	4.8 (1.90)	5.1 (1.82)	0.26	4.9 (1.83)	4.5 (2.09)	5.0 (2.09)	4.6 (1.50)	0.54	4.8 (1.80)	5.0 (1.96)	0.31
Maintaining social distance	2.4 (1.47)	2.4 (1.47)	2.1 (1.49)	0.17	2.3 (1.51)	2.4 (1.37)	0.84	2.3 (1.43)	2.4 (1.56)	2.4 (1.63)	2.4 (1.54)	>0.99	2.4 (1.49)	2.3 (1.46)	0.58
**Cue to action**															
Hand washing	6.7 (0.57)	6.7 (0.58)	6.7 (0.55)	0.93	6.8 (0.48)	6.5 (0.75)	0.003^*^	6.7 (0.52)	6.8 (0.42)	6.6 (0.83)	6.5 (0.70)	0.20	6.7 (0.60)	6.7 (0.55)	0.88
Face mask wearing	5.3 (1.75)	5.3 (1.73)	5.3 (1.83)	0.91	5.4 (1.64)	5.1 (2.00)	0.13	5.3 (1.76)	5.5 (1.60)	5.7 (1.53)	4.6 (2.19)	0.12	5.3 (1.79)	5.3 (1.71)	0.85
Maintaining social distance	6.4 (0.97)	6.4 (0.97)	6.4 (0.95)	0.93	6.5 (0.87)	6.2 (1.18)	0.03^*^	6.4 (0.87)	6.5 (0.91)	6.6 (0.89)	5.9 (1.85)	0.07	6.5 (1.03)	6.4 (0.90)	0.49
**Self-efficacy**															
Hand washing	6.5 (0.87)	6.5 (0.74)	6.3 (1.25)	0.19	6.5 (0.89)	6.4 (0.83)	0.54	6.5 (0.79)	6.5 (1.09)	6.4 (1.16)	6.7 (0.45)	0.61	6.6 (0.68)	6.4 (1.03)	0.01^*^
Face mask wearing	3.4 (1.98)	3.4 (1.96)	3.5 (2.04)	0.86	3.3 (1.95)	3.8 (2.04)	0.06	3.5 (1.93)	3.2 (1.99)	3.7 (2.17)	2.8 (2.01)	0.39	3.6 (1.97)	3.2 (1.97)	0.13
Maintaining social distance	6.2 (1.15)	6.2 (1.07)	6.0 (1.39)	0.17	6.2 (1.16)	6.1 (1.12)	0.37	6.1 (1.18)	6.3 (1.01)	6.4 (0.87)	5.9 (1.56)	0.26	6.3 (0.95)	6.0 (1.32)	0.02^*^

† *1 as extremely low; 7 as extremely high*.

* *p < 0.05*.

Respondents reported a medium level of pandemic worry severity (*M* = 4.2, *SD* = 1.92). Females reported a significantly higher worry severity compared to males (4.4 vs. 3.7, *p* = 0.004). No differences between worry severity were found among students of different ages, races, and academic settings (Table 2). For the pandemic worry frequency in the past week, 51% of respondents reported that they worried about getting COVID-19 less than half the time and 37% of respondents reported more than half the time. Specifically, 7% of respondents reported always worried about getting COVID-19 in the past week (Table 1).

Hand washing was positively correlated with face mask wearing (*r* = 0.14, *p* = 0.01) and maintaining social distance (*r* = 0.26, *p* < 0.001). However, face mask wearing was not correlated with maintaining social distance (*r* = 0.10, *p* = 0.07). Respondents reported that they were extremely likely to wash their hands (*M* = 6.8, *SD* = 0.48) and maintain social distance (*M* = 6.6, *SD* = 0.92), but were moderately unlikely to wear a face mask in public (*M* = 2.2, *SD* = 1.51). Females were significantly more likely to wash hands compared to males (6.9 vs. 6.7, *p* = 0.006). Asian Americans were significantly more likely to wear a face mask compared to other racial groups (Caucasian: 2.1, African American: 2.0, Asian American: 3.0, Other: 2.1, *p* = 0.004; Table 2).

Respondents reported a slightly high perceived susceptibility to COVID-19 (*M* = 4.7, *SD* = 1.79). The perceived susceptibility was different among different racial groups (Caucasian: 4.8, African American: 4.6, Asian American: 4.0, Other: 5.5, *p* = 0.006) and those attended didactic course perceived a significantly lower susceptibility compared to those engaged in experiential learning (4.4 vs. 5.1, *p* < 0.001). Respondents reported a moderately high perceived severity of COVID-19 (*M* = 5.8, *SD* = 1.13). Perceived benefits were moderately high for hand washing (*M* = 6.1, *SD* = 1.01) and maintaining social distance (*M* = 6.2, *SD* = 0.97), but were medium for face mask wearing (*M* = 3.5, *SD* = 1.71). Perceived barriers were slightly high for face mask wearing (*M* = 4.9, *SD* = 1.88), slightly low for hand washing (*M* = 3.4, *SD* = 2.04), and moderately low for maintaining social distance (*M* = 2.4, *SD* = 1.47). Cue to action was extremely high for hand washing (*M* = 6.7, *SD* = 0.57), moderately high for maintaining social distance (*M* = 6.4, *SD* = 0.97), and slightly high for face mask wearing (*M* = 5.3, *SD* = 1.75). Females reported a significantly higher cue to action of hand washing (6.8 vs. 6.5, *p* = 0.003) and a significantly higher cue to action of maintaining social distance (6.5 vs. 6.2, *p* = 0.03) compared to males. Self-efficacy was extremely high for hand washing (*M* = 6.5, *SD* = 0.87), moderately high for maintaining social distance (*M* = 6.2, *SD* = 1.15), and slightly low for face mask wearing (*M* = 3.4, *SD* = 1.98). Those attended didactic course reported a significantly higher self-efficacy of hand washing (6.6 vs. 6.4, *p* = 0.01) and a significantly higher self-efficacy of maintaining social distance (6.3 vs. 6.0, *p* = 0.02) compared to those engaged in experiential learning (Table 2).

Perceived severity (β: 0.08; 95% CI: 0.04–0.13) and cue to action (β: 0.21; 95% CI: 0.11–0.30) were predictors for hand washing. Being a male was negatively associated with hand washing (β: −0.13; 95% CI: −0.24 to −0.01). Determinants of face mask wearing included pandemic worry (β: 0.18; 95% CI: 0.10–0.26), perceived benefits (β: 0.29; 95% CI: 0.21–0.38), cue to action (β: 0.10; 95% CI: 0.01–0.18), self-efficacy (β: 0.18; 95% CI: 0.11–0.25), and being of an Asian American (β: 0.57; 95% CI: 0.17–0.97). Perceived barriers were negatively associated with face mask wearing (β: −0.12; 95% CI: −0.20 to −0.05). Cue to action (β: 0.38; 95% CI: 0.28–0.49) and self-efficacy (β: 0.13; 95% CI: 0.04–0.23) were predictors for maintaining social distance (Table 3).

**Table 3:** Factors associated with preventive health behaviors.

	**Hand washing**			**Face mask wearing**			**Maintaining social distance**		
	**β**	**95% CI**		**β**	**95% CI**		**β**	**95% CI**	
**Pandemic worry**	−0.01	−0.03	0.02	0.18	0.10	0.26	0.04	−0.01	0.09
**Knowledge**	0.02	−0.03	0.06	−0.03	−0.16	0.11	−0.02	−0.10	0.07
**Health belief model**									
Perceived susceptibility	0.03	0.00	0.06	−0.01	−0.09	0.07	0.03	−0.03	0.08
Perceived severity	0.08	0.04	0.13	−0.04	−0.18	0.09	0.07	−0.01	0.16
Perceived benefits	0.03	−0.02	0.08	0.29	0.21	0.38	−0.03	−0.13	0.06
Perceived barriers	0.00	−0.03	0.02	−0.12	−0.20	0.05	−0.06	−0.13	0.00
Cue to action	0.21	0.11	0.30	0.10	0.01	0.18	0.38	0.28	0.49
Self-efficacy	0.06	0.00	0.12	0.18	0.11	0.25	0.13	0.04	0.23
**Demographics**									
**Age**									
18–26	Ref			Ref			Ref		
27+	−0.05	−0.17	0.08	−0.01	−0.35	0.34	−0.02	−0.24	0.20
**Gender**									
Female	Ref			Ref			Ref		
Male	−0.13	−0.24	−0.01	0.21	−0.11	0.53	0.02	−0.18	0.23
**Ethnicity**									
Caucasian	Ref			Ref			Ref		
African American	0.10	−0.05	0.25	−0.09	−0.50	0.32	−0.11	−0.37	0.16
Asian American	0.01	−0.14	0.15	0.57	0.17	0.97	0.13	−0.13	0.39
Other	0.18	−0.03	0.39	0.03	−0.56	0.61	0.21	−0.16	0.59
**Academic setting**									
Didactic course	Ref			Ref			Ref		
Experiential learning	0.02	−0.08	0.12	0.00	−0.28	0.28	−0.10	−0.28	0.08

## Discussion

This study found that student pharmacists had a medium level of pandemic worry severity and females had a higher worry severity than males. Pandemic worry might be associated with adverse psychological outcomes and students are more vulnerable. Cao et al. (7) found that 25% of college students reported experiencing anxiety amidst the COVID-19 outbreak in China. Wang et al. (8) reported that moderate or severe psychological impact was experienced by 54% of the general population in China. Females and students were significant predictors of experiencing greater psychological impact amidst the COVID-19 outbreak (8). Student pharmacists might be more psychologically vulnerable due to their younger age, the challenges they face with the transition into adulthood and to the profession (e.g., managing finances, adapting to new learning regimes, making new friends, and creating a new identity as a pharmacist). The abrupt change in their daily lives associated with strict government measures to contain the outbreak (e.g., school and non-essential business closures), the consequences of these measures (e.g., financial pressure to pay tuition expenses amidst uncertain employment prospect and social isolation from friends), and the rapid changes in the social and economic climate during the COVID-19 outbreak, might further compound their pandemic worry.

Additional psychosocial and economic factors that disproportionately affect females, such as low social status, income inequality, gender-based violence, and caregiving responsibility, might explain the higher level of pandemic worry observed (18). Potential protective factors of pandemic worry during the COVID-19 outbreak include social support and living with parents (7). Therefore, strategies such as creating virtual peer support groups, organizing virtual activities, and encouraging students to maintain contact with their family and friends virtually, might help to mitigate the psychological impact of COVID-19. If appropriate and feasible, student pharmacists might also consider moving back with their parents during the COVID-19 outbreak.

Student pharmacists reported their knowledge of COVID-19 to be slightly high. Improvement of knowledge, dissemination of additional health information, provision of regular updates on the latest information, and accessibility of information *via* reliable sources, have been identified as protective factors against adverse psychological outcomes during the COVID-19 outbreak (8). Websites and social media proved to be effective strategies for getting knowledge to student pharmacists based on our results. Therefore, increasing education and providing regular updates through government websites or social media accounts could be used to mitigate the psychological impact associated with COVID-19.

Student pharmacists reported that they were extremely likely to wash their hands and maintain social distance, but were moderately unlikely to wear a face mask. Given the low willingness to wear a face mask, predictors identified in our study should be considered to encourage face mask wearing. Components of the HBM that predicted face mask wearing were perceived benefits, perceived barriers, cue to action, and self-efficacy. Perceived benefits and cue to action as predictors of face mask wearing were consistent with previous studies conducted in Asia (10–12). Barriers to face mask wearing identified in previous studies included discomfort (e.g., difficulty with breathing), inconvenience (e.g., need to remove masks during meal times), forgetfulness, and social acceptance (19). Strategies should be taken to improve face mask wearing following the recent CDC guidelines revision. For example, government, public health officials, and healthcare professionals can actively promote face mask wearing, as well as improve the public's knowledge on the effectiveness of face masks in reducing COVID-19 transmission. By widely promoting face mask wearing, this can increase social acceptance and reduce stigmatization and discrimination against people who wear them (20). Messaging should also make clear of the recommendations for the use of cloth masks and to not use medical masks reserved for medical professionals (17). Videos on how to make cloth masks can be created by public health officials or organizations and be uploaded on official government websites and YouTube, in order to improve self-efficacy of face mask use. YouTube, due to its considerable reach, has recently been identified as an under-tapped platform for promoting preventive health behaviors amidst the COVID-19 pandemic (21). As this study also identified cue to action as a predictor of hand washing and maintaining social distance, public messaging should continue to emphasize the importance of hand washing and maintaining social distancing, along with face mask wearing, in containing the spread of COVID-19.

There are several strengths of this study. First, this is the first study evaluating pandemic worry among student pharmacists. The results of this study highlighted the vulnerability of student pharmacists to the psychological impact of the pandemic and emphasize the need to implement strategies to mitigate this effect. Second, predictors of face mask wearing could be used to improve face mask use among the public in light of recent expansion in CDC guidelines. Third, this study applied the theoretical framework of the health belief model to systematically assess the determinants of preventive health behaviors. This could inform public health initiatives to contain the COVID-19 outbreak when universities and colleges reopen for onsite learning. This study has some limitations worth noting. First, the survey was conducted prior to CDC modifying recommendations on face mask wearing. Respondents' perception of face mask wearing reported might change in light of the new recommendations. Second, the survey collected information at a single point in time. Thus, it cannot capture changes in the students' pandemic worry and preventive health behaviors as the COVID-19 pandemic evolves. Third, this study relied on students' self-reports using a survey. It might not reflect their actual level of pandemic worry and preventive health behaviors due to social desirability. Fourth, this study only included student pharmacists in the study population. The results might not be generalizable to other health professional students.

## Conclusion

The COVID-19 pandemic has generated a medium-level of pandemic worry among student pharmacists in the United States. Females reported a higher level of pandemic worry than males. Strategies should be implemented to reduce the psychological impact of COVID-19 pandemic. For preventive health behaviors, student pharmacists reported that they were moderately unlikely to use a face mask and were extremely likely to engage in hand washing and social distancing. The health belief model components that predicted face mask wearing (i.e., perceived benefits, perceived barriers, cue to action, and self-efficacy) should be incorporated in efforts to improve face mask wearing following CDC's recent expansion of face mask recommendations. Continued monitoring of pandemic worry and preventive health behaviors among student pharmacists are of great significance when universities and colleges are preparing for onsite learning.

## Data Availability Statement

The raw data supporting the conclusions of this article will be made available by the authors, without undue reservation.

## Ethics Statement

The studies involving human participants were reviewed and approved by University of Tennessee Health Science Center. The patients/participants provided their written informed consent to participate in this study.

## Author Contributions

ML and GL: conceptualization, methodology, and writing. SH: methodology and writing. RS: methodology and analysis. JY: conceptualization, methodology, analysis, writing, and supervision. All authors contributed to the article and approved the submitted version.

## Conflict of Interest

The authors declare that the research was conducted in the absence of any commercial or financial relationships that could be construed as a potential conflict of interest.
